# Relationship between Serum Vitamin D and Calcium Levels and Vitamin D Receptor Gene Polymorphisms in Colorectal Cancer

**DOI:** 10.1155/2019/8571541

**Published:** 2019-08-26

**Authors:** Ayat B. Al-Ghafari, Khadijah S. Balamash, Huda A. Al Doghaither

**Affiliations:** ^1^Biochemistry Department, Faculty of Science, King Abdulaziz University, Jeddah, Saudi Arabia; ^2^Cancer Metabolism and Epigenetics Unit, Faculty of Science, King Abdulaziz University, Jeddah, Saudi Arabia; ^3^Experimental Biochemistry Unit, King Fahd Medical Research Centre, King Abdulaziz University, Jeddah, Saudi Arabia; ^4^Cancer and Mutagenesis Unit, King Fahd Medical Research Centre, King Abdulaziz University, Jeddah, Saudi Arabia

## Abstract

**Background:**

Many epidemiological studies have shown that vitamin D deficiency is associated with various types of human cancers. The biological action of vitamin D and its metabolites is mediated by the transcription factor vitamin D receptor (VDR). The* VDR* gene is highly expressed in the colon and is involved in many biological functions. The aim of the current study was to assess the relationship between serum vitamin D metabolite and calcium levels with* VDR *polymorphisms in normal and colorectal cancer (CRC) patients.

**Methods:**

Fifty Saudi CRC patients and fifty controls were enrolled in the study. The levels of total vitamin D, 25(OH)D_3_, and calcium were measured in serum.

**Results:**

The homozygous genotype (aa) of the* ApaI VDR* polymorphism (rs7975232) was found to correlate with total serum vitamin D levels of CRC patients, while the heterozygous (Tt)* TaqI VDR* polymorphism (rs731236) was associated with serum calcium levels. In contrast, the* BsmI* and* FokI VDR* polymorphisms (rs1544410 and rs2228570, resp.) did not affect the serum levels of total vitamin D, 25-hydroxyvitamin D_3_, and calcium.

**Conclusion:**

Appropriate vitamin D levels were shown to be important in preventing the onset of CRC.

## 1. Introduction

Vitamin D is an effective regulator of several physiologic processes, such as calcium (Ca) homeostasis and innate and adaptive immunity [1, 2]. On a molecular level, the effects of vitamin D are mediated by the vitamin D receptor (VDR) [3, 4]. The VDR is a transcription factor and a member of the nuclear receptor superfamily [5]. Epidemiological and experimental studies reveal that the VDR and its ligands are promising targets for the prevention and possible treatment of cancer, autoimmune diseases, and infections, as well as of bone and mineral disorders [6, 7].

Serum levels of 25-hydroxyvitamin D [25(OH)D] induced by exposure to sunlight never exceed 60 ng/mL (149.76 nmol/L). According to the Mayo medical laboratories reference ranges, the total serum level of 25(OH)D ranges from 25 to 80 ng/ml, with a 25(OH)D level of less than 30 ng/ml defined as vitamin D insufficiency, a 25(OH)D level of less than 20 ng/ml defined as vitamin D deficiency, and a 25(OH)D level of 80 ng/ml or greater defined as possible toxicity [8]. Interindividual differences in the human genome, often referred to as gene polymorphisms, are called variants when they appear in at least 1% of the population. A number of biological processes and diseases, such as Alzheimer's disease, autism, type 2 diabetes mellitus, obesity, and rheumatoid arthritis, associate with* VDR* polymorphisms [5, 9–12]. However, the molecular mechanism by which the variants of the* VDR* gene exert these effects is unclear [1, 13]. This also applies to the role of* VDR* polymorphisms in colorectal cancer (CRC).

According to the 14^th^ cancer incidence report issued by the Saudi Cancer Registry in 2014, in the Kingdom of Saudi Arabia, CRC represents 10.4% of all cancers and is the second most common cancer type after breast cancer among Saudi cancer patients. Among Saudi males, CRC is the most common cancer (11.8%) [14, 15]. Compared to other types of cancers, CRC occurs sporadically [16] and presents at a younger age in Saudis, especially in women [14]. The underlying etiology of CRC is not well determined, but it has been proposed that the onset of CRC results from the interaction between exogenous chemical and biological factors such as age, diet, smoking, physical activity, and individual genetic predisposition [17, 18].

Available treatment options for CRC depend on the stage of the disease. Treatment of CRC has systematically advanced over the past few years with the introduction of effective chemotherapeutic agents. These drugs are often used in combination with biological therapy in patients with advanced disease, such as enhancing the response of immune system and the use of minerals and vitamins [19]. A number of studies have investigated the contribution of vitamin D status and* VDR* gene variants (polymorphisms and mutations) in several types of cancer, including CRC. Most of these studies showed that insufficient vitamin D status may contribute to CRC development [20–24]. However, the results of studies on* VDR* gene polymorphisms and their relation to CRC development and prognosis are contradictory [25–28]. In fact, these correlations mostly result from the interaction between* VDR *polymorphisms and other factors, such as Ca and vitamin D intake, plasma levels of 1,25-dihydroxyvitamin D_3_ [1,25(OH)_2_D_3_], UV radiation exposure, obesity, and energy intake [29–32].The aim of this study was to analyze the relationship between serum total vitamin D, 25(OH)D_3_, and Ca levels and* VDR* polymorphisms in the manifestation of CRC.

## 2. Materials and Methods

### 2.1. Subjects

Fifty CRC patients (12 females and 38 males) aged 30–80 years were selected from the day care unit of King Abdulaziz University Hospital (Jeddah, Saudi Arabia). The same number of age- and sex-matched healthy controls were randomly selected from the blood bank unit. All study participants were of Saudi ethnicity and signed an ethical consent form, following the Declaration of Helsinki's human ethical principles. CRC patients were accepted at any stage of the disease but had no primary tumors other than CRC. Members of the control group had no previous history of any systemic illness or cancer. The study protocol as well as the questionnaire and the informed consent were approved by the ethics committee of King Abdulaziz University, Faculty of Medicine (reference no. 379-17). From each study participant, two blood samples were drawn for DNA and serum extraction.

### 2.2. Genotyping of Vitamin D Receptor (VDR) Variants

Extracted genomic DNA (100 ng/ml) was amplified in a 25 *µ*l polymerase chain reaction (PCR) with 12.5 *µ*l HotStart-IT® FideliTaq™ PCR Master Mix (2X) (Affymetrix/USB™, 71156, USA), 9.5 *µ*l RNase free water (Affymetrix/ USB™, 7732-18-5, USA), and 1 *µ*l of each primer (0.2 *µ*mol). The primers and the PCR thermocycler reactions were previously published in Hajj* et al.* [29] for* ApaI* and* BsmI*, whereas for* TaqI* and* FokI* in Rizk* et al.,* [21]. The amplified PCR products were genotyped with restriction fragment length polymorphism (RFLP) using appropriate restriction endonucleases from Thermo Fisher Scientific (Waltham, MA, USA) [FastDigest* ApaI* (FD1414), FastDigest* TaqI*, (FD0674), FastDigest* Mva1269I* (FD0964), and FastDigest* FokI* (FD2144)] following the manufacturer's instructions. The different genotypes for each variant were confirmed with 2% agarose gel electrophoresis and 10% of the samples were selected randomly for further DNA sequencing confirmation.

### 2.3. Measurement of Serum Total Vitamin D, 25(OH)*D*_3_, and Ca Levels

The level of total vitamin D [25(OH)D_2_ and 25(OH)D_3_] in serum was measured using a commercial LIAISON®25OH Vitamin D Total Assay Kit (DiaSorin, USA). Serum Ca and 25(OH)D_3_ levels were measured by the ADVIA Centaur chemiluminescent reaction in the Biochemistry laboratory at King Abdulaziz University Hospital.

### 2.4. Statistical Analysis

All statistical analyses were performed on GraphPad Prism version 7.00 (San Diego, California, USA). The Mann-Whitney test was used to compare differences between two independent groups with either one physical or biochemical variable. The Kruskal-Wallis test with Dunn's multiple comparison was applied to correlate the genotype distribution of each* VDR* variant with total vitamin D, 25(OH)D_3_, and Ca levels measured in the serum.* P *values < 0.05 were considered statistically significant. Values in tables were represented as mean ± standard error of mean (SEM).

## 3. Results

A total of 100 study participants were enrolled: fifty CRC patients and fifty healthy controls. All CRC patients were treatment-experienced and had completed the full course of chemotherapy. The common chemotherapy regimens of the CRC cases were capecitabine (Xeloda) as neoadjuvant therapy with radiation; oxaliplatin and capecitabine (Xelox) intravenously for 6 to 8 weeks; and, finally, irinotecan intravenously and capecitabine as tablets twice a day (Xeliri) for 2 to 3 weeks. The baseline of sociodemographic and laboratory measurements for study participants is presented in Table 1. There was a highly significant difference in body mass index (BMI) between CRC patients and controls (*P *< 0.0001). This is due to poor food intake in the patients as a consequence of chemotherapy, resulting in a loss of appetite. Table 1 shows highly significant differences in serum total vitamin D and Ca levels between patients and controls (*P* < 0.0001). In contrast, serum 25(OH)D_3_ levels were not significantly different between the two groups (*P* > 0.05).

In order to find a possible correlation between the different genotypes of the* VDR* gene polymorphisms (*ApaI*,* TaqI*,* BsmI*, and* FokI)* and the serum total vitamin D, 25(OH)D_3_, and Ca levels, a Kruskal-Wallis test was performed (Tables 2–5). In the case of the* ApaI* polymorphism, only CRC patients with homozygous* ApaI* (aa) genotypes showed a higher level of total vitamin D in the serum compared to other genotypes (AA) and (Aa) (*P* value = 0.0071) (Table 2). In contrast, in healthy controls, no significant correlation with any* ApaI *genotype was found. However, the serum 25(OH)D_3_ level in both CRC patients and controls did not significantly correlate with the genotypes of* ApaI*. Interestingly, Ca levels showed a significant correlation with the heterozygous (Aa) genotype in the CRC patient group (the level of Ca was lower in patients with the Aa genotype compared with genotypes AA and aa patients,* P *= 0.04). In the case of* TaqI* polymorphism, the values of the three biochemical tests did not show any significant correlation with* TaqI* genotypes (Table 3). However, heterozygous (Tt) and homozygous (tt) CRC patients had lower total vitamin D levels compared to CRC patients with a normal genotype (TT). In addition, neither the heterozygous nor the homozygous genotypes of* BsmI* (Table 4) or of* FokI* (Table 5) correlated significantly with serum total vitamin D, 25(OH)D_3_, and Ca levels (*P* > 0.05). However, there was a significant reduction in serum 25(OH)D_3_ levels in healthy controls with the homozygous (ff)* FokI* genotype (*P *= 0.02).

## 4. Discussion

CRC is one of the most aggressive cancers and is responsible for many cancer-related deaths in Saudi Arabia [14]. Although many advances have been made to identify CRC in the early stages, most patients suffering from this cancer are diagnosed at late stages with severe symptoms. Many studies have tried to identify proteins and signaling pathways which may serve as prognostic markers for CRC and other cancer types. One of them is the determination of the genetic signature of the* VDR* gene polymorphisms in combination with vitamin D status [20–28]. In this study, the genotype distribution of the four* VDR* gene polymorphisms in CRC patients and healthy controls were compared with serum total vitamin D, 25(OH)D_3_, and Ca levels. The levels of both total vitamin D and Ca in the blood of CRC patients were found to be significantly lower than in healthy controls. On the other hand, the level of 25(OH)D_3_ did not show any significant difference between the two groups. Our results agreed with other published clinical data that showed a lower vitamin D status [total or 25(OH)D_3_] in prostate, breast, and colon cancer patients compared to healthy subjects. However, the exact mechanism behind these associations remains unclear [33–35].

A recent study of CRC patients before and after receiving chemotherapy suggests that vitamin D repletion is a feasible intervention during chemotherapy [36]. It found that, among patients with a new diagnosis of CRC (no chemotherapy course has started), most patients have deficient or insufficient levels of 25(OH)D_3_, whereas in patients who received chemotherapy with vitamin D supplementation, the serum 25(OH)D_3_ levels increased. Another study performed on CRC patients showed that higher circulating vitamin D was related to a significant effect on lowering CRC risk in females but not in males [13]. A clinical trial study performed on stage II and stage III CRC patients evaluated the effect of vitamin D status and physical activity as an intervention method for CRC prevention and treatment [30]. They found that CRC patients of all stages with levels of vitamin D in the highest quantiles had improved overall survival rates compared to those with levels in the lowest quantiles. Our study showed that the homozygous (aa) version of the* ApaI VDR* polymorphism correlated with total vitamin D levels but not with 25(OH)D_3_ concentrations in CRC patients. Most previous studies have correlated the level of vitamin D with the risk of CRC or have studied the effect of* VDR* gene polymorphisms on developing CRC. Little is known about how these polymorphisms affect the levels of circulating vitamin D, 25(OH)D_3_, and Ca [37]. In contrast to our study, 25(OH)D_3_ levels in healthy Indians showed a significant association for the* TaqI* but not the* FokI VDR* polymorphism [38].

In this study, serum Ca levels were found to correlate significantly with the heterozygous (Tt) genotype of the* TaqI VDR* polymorphism but not with the other three* VDR* variants tested. It is known that Ca levels are usually affected in cancer patients and are related to the calcium sensing receptor (CaSR) and to vitamin D metabolism. Fuszek* et al.* [39] found a lower level of Ca in CRC patients with normal 25(OH)D_3_ levels. Ca levels inversely correlated with the level of the cancer antigen 19-9 (CA 19-9) tumor marker but not with the carcinoembryonic antigen (CEA) or alpha fetoprotein (AFP) tumor markers or the CaSR genotypes. A study with 922 Korean CRC patients showed that Ca consumption was inversely related to CRC risk [40]. Another recent study suggested that lower serum Ca levels are correlated with Nigerian CRC patients that show high CEA levels compared to patients with low levels of the tumor marker [41].

## 5. Conclusion

This study found that the homozygous genotype (aa) of the* VDR* SNP* ApaI* correlates with total vitamin D level in the serum of CRC patients and that the heterozygous genotype (Tt) of the* VDR *SNP* TaqI* significantly associates with serum Ca levels. Thus, our findings found that vitamin D status may play an important role in CRC tumorigenesis. However, more studies on larger number of CRC patients in Saudi Arabia or on cancerous tissues are needed to elucidate further the current findings and study the molecular mechanisms.

## Figures and Tables

**Table 1:** Socio-demographic and biochemical analysis of patients and controls.

Physical and biochemical	Patients (n=50)	Controls (n=50)	*P* value
characteristics	Mean±SEM	Mean±SEM	
Age (years)	55.58±1.779	51.72±1.597	0.06
Height (cm)	165.6±1.372	163.8±1.686	0.57
Weight (kg)	74.96±2.164	80.80±2.216	0.10
BMI (kg/m^2^)	27.26±0.776	30.20±0.761	0.02
Hip (cm)	111.8±2.749	108.0±2.396	0.40
Waist (cm)	101.1±2.906	101.8±3.197	0.73
Waist-to-hip ratio	0.912±0.021	0.946±0.023	0.79
Total vitamin D (nmol/L)	88.22±9.56	143.30±10.33	< 0.0001
25(OH)D_3_ (nmol/L)	33.16±1.989	37.64±2.027	0.10
Ca (mmol/L)	2.22±0.021	2.32±0.014	< 0.0001

BMI: Body mass index, Ca: Calcium, *P* value was calculated by Mann-Whitney test.

**Table 2:** Correlation between different genotypes of *ApaI *SNP with the expression of total vitamin D, 25(OH)D_3_, and calcium serum levels in patients and controls.

*Biochemical test*	*Patients (n=50)*			*Controls (n=50)*			*P value*
	*Frequency (*%)			*Frequency (*%)			
	*AA*	*Aa*	*aa*	* AA*	*Aa*	*aa*	
	22 (44%)	19 (38%)	9 (18%)	37 (74%)	8 (16%)	5 (10%)	
Total vitamin D (nmol/L)	67.96±5.84	73.58±10.68	171.60±35.82	143.80±12.88	132.30±19.71	155.20±28.08	< 0.0001
*P* value	0.0071			0.86			

25(OH)D_3_ (nmol/L)	27.95±2.19	37.63±3.05	36.44±6.70	37.68±2.06	33.13±5.28	44.60±11.06	0.12
*P* value	0.08			0.81			

Calcium (mmol/L)	2.26±0.021	2.15±0.042	2.26±0.044	2.31±0.016	2.34±0.023	2.33±0.055	0.0007
*P* value	0.04			0.47			

*P* value was calculated by Kruskal-Wallis test with Dunn's multiple comparison.

**Table 3:** Correlation between different genotypes of *TaqI *SNP with the expression of total vitamin D, 25(OH)D_3_, and calcium serum levels in patients and controls.

*Biochemical test*	*Patients (n=50)*			*Controls (n=50)*			*P value*
	*Frequency (*%)			*Frequency (*%)			
	*TT*	*Tt*	*tt*	*TT*	*Tt*	*tt*	
	24 (48%)	20 (40%)	6 (12%)	46 (92%)	3 (6%)	1 (2%)	
Total vitamin D (nmol/L)	106.20±18.12	71.87±8.06	72.51±9.89	143.10±10.92	134.90±45.63	168.10±0.0	0.0008
*P* value	0.76			0.83			

25(OH)D_3_ (nmol/L)	36.29±3.21	29.35±2.59	33.33±5.49	38.04±2.14	34.33±8.84	29.00±0.0	0.39
*P* value	0.38			0.74			

Calcium (mmol/L)	2.23±0.022	2.19±0.044	2.26±0.023	2.31±0.014	2.30±0.051	2.40±0.0	0.0042
*P* value	0.54			0.51			

*P* value was calculated by Kruskal-Wallis test with Dunn's multiple comparison.

**Table 4:** Correlation between different genotypes of *BsmI *SNP with the expression of total vitamin D, 25(OH)D_3_, and calcium serum levels in patients and controls.

*Biochemical test*	*Patients (n=50)*			*Controls (n=50)*			*P value*
	*Frequency (*%)			*Frequency (*%)			
	*BB*	*Bb*	*bb*	*BB*	*Bb*	*bb*	
	20 (40%)	22 (44%)	8 (16%)	6 (12%)	28 (56%)	16 (32%)	
Total vitamin D (nmol/L)	107.40±16.58	79.32±14.77	69.30±9.71	124.0±23.87	130.4±14.40	172.4±16.54	< 0.0001
*P* value	0.25			0.07			

25(OH)D_3_ (nmol/L)	36.50±3.85	30.77±2.58	31.38±3.27	36.17±4.60	38.86±2.46	36.06±4.45	0.48
*P* value	0.58			0.79			

Calcium (mmol/L)	2.23±0.021	2.18±0.041	2.27±0.035	2.34±0.013	2.31±0.021	2.31±0.023	0.0031
*P* value	0.35			0.57			

*P* value was calculated by Kruskal-Wallis test with Dunn's multiple comparison.

**Table 5:** Correlation between different genotypes of *FokI *SNP with the expression of total vitamin D, 25(OH)D_3_, and calcium serum levels in patients and controls.

*Biochemical test*	*Patients (n=50)*			*Controls (n=50)*			*P value*
	*Frequency (*%)			*Frequency (*%)			
	*FF*	*Ff*	*ff*	* FF*	*Ff*	*ff*	
	33 (66%)	13 (26%)	4 (8%)	24 (48%)	23 (46%)	3 (6%)	
Total vitamin D (nmol/L)	91.62±13.49	79.15±11.75	91.89±26.26	154.0±16.46	136.20±14.21	109.0±11.36	0.0013
*P* value	0.87			0.57			

25(OH)D_3_ (nmol/L)	33.00±2.69	32.69±3.00	36.00±6.88	42.83±2.62	33.48±2.98	28.00±9.02	0.06
*P* value	0.82			0.02			

Calcium (mmol/L)	2.22±0.029	2.21±0.023	2.19±0.058	2.33±0.018	2.31±0.019	2.22±0.095	0.0012
*P* value	0.39			0.30			

*P* value was calculated by Kruskal-Wallis test with Dunn's multiple comparison.

## Data Availability

The data used to support the findings of this study are available from the corresponding author upon request.
